# Fluoride Concentration of Drinking Waters and Prevalence of Fluorosis in Iran: A Systematic Review

**DOI:** 10.5681/joddd.2013.001

**Published:** 2013-02-21

**Authors:** Saber Azami-Aghdash, Morteza Ghojazadeh, Fatemeh Pournaghi Azar, Mohammad Naghavi-Behzad, Mostafa Mahmoudi, Zahra Jamali

**Affiliations:** ^1^Tabriz Health Services Management Research Center, Tabriz University of Medical Sciences, Tabriz, Iran; ^2^Associate Professor, Liver and Gastrointestinal Disease Research Center, Tabriz University of Medical Sciences, Tabriz, Iran; ^3^Assistant Professor, Department of Operative Dentistry, Faculty of Dentistry, Tabriz University of Medical Sciences, Tabriz, Iran; ^4^Medical Philosophy and History Research Center, Tabriz University of Medical Sciences, Tabriz, Iran; ^5^Assistant Professor, Department of Oral & Maxillofacial Pathology, Faculty of Dentistry, Birjand University of Medical Sciences, Birjand, Iran; ^6^Dental and Periodontal Research Center, Tabriz University of Medical Sciences, Tabriz, Iran; ^7^Assistant Professor, Department of Oral Medicine, Faculty of Dentistry, Tabriz University of Medical Sciences, Tabriz, Iran

**Keywords:** Concentration, drinking waters, fluoride, fluorosis, prevalence

## Abstract

**Background and aims:**

The aim of the present study was to systematically review fluoride concentration of drinking waters and prevalence of fluorosis in Iran through systematically evaluating results of studies conducted in this regard.

**Materials and methods:**

In this systematic review study, the required data was collected using keywords including drinking water fluoride, fluoride concentration, Fluorosis, dent*, Iran*, and their Persian equivalents through PubMed, ScienceDirect, IranMedex, SID, MEDLIB, and Magiran databases. Out of 617 articles, 29 articles were finally considered after excluding the remaining articles which were not related to the study objectives. Following precise studying and extraction, the relevant data were summarized in extraction tables and analyzed manually. Excel 2007 software was used to draw diagrams.

**Results:**

4434 samples of surface, ground, and tap water resources collected within 236 months during all seasons in 17 provinces of Iran were used in 29 articles determining fluoride concentrations of drinking water. Average fluoride concentration was estimated to be 0.43 ± 0.17 ppm with zero and 3.06 as minimal and maximal values. The least concentration was seen in tap water. Fluoride concentration of only three provinces was in accordance with the global standard. According to estimations, prevalence of fluorosis was 61% with only 1% as severe fluorosis.

**Conclusion:**

Despite lower than standard concentrations of fluoride in drinking water, a relatively high level of fluorosis was seen in Iran.

## Introduction

At present, fluoride concentration of drinking water and the dental caries are regarded as one of the most common health problems and main concerns of dentists,^1^ since low fluoride concentration of the consumed water, i.e. less than the standard rate (1.2-6 ppm), results in caries,^2^ and if progressed, fluorosis.^3^ According to World Health Organization, standard rate of fluoride of drinking water is 0.5–1 ppm.^4^ Studies conducted at different parts of the world reported variable concentrations of water fluoride and fluorosis,^5-7^ such that it was 0.19 ppm in South Africa study with prevalence rate of 47% for fluorosis.^3^ According to World Health Organization, highest rate of prevalence of fluorosis is seen in China and India.^6^ Studies conducted in Iran reported different fluoride concentrations of water and prevalence of fluorosis.^8-11^ Although different policies including adding of fluoride to drinking water, use of fluoride-contained toothpastes and mouthwashes are made when there is insufficient fluoride concentration in drinking waters, there is controversies among experts in this regard.^2,4^ Standard value of water fluoride varies according to ecological and social conditions.^5,8^ In Iran, standard values should be identified for every region considering their ecological conditions since Iran has different weather and the temperature varying +50°C to −20°C in some regions.^8^ Although there are several studies conducted at different parts of Iran, there is not any comprehensive study evaluating fluoride concentration of waters of different resources and prevalence of fluorosis, according to results of articles review. Therefore, the present study aimed at systematically evaluating the studies published on fluoride concentration of different water sources as well as prevalence of fluorosis and providing a clear and comprehensive viewpoint from status of fluoride found in drinking waters and prevalence rate of fluorosis.

## Materials and Methods

**Figure 1:**
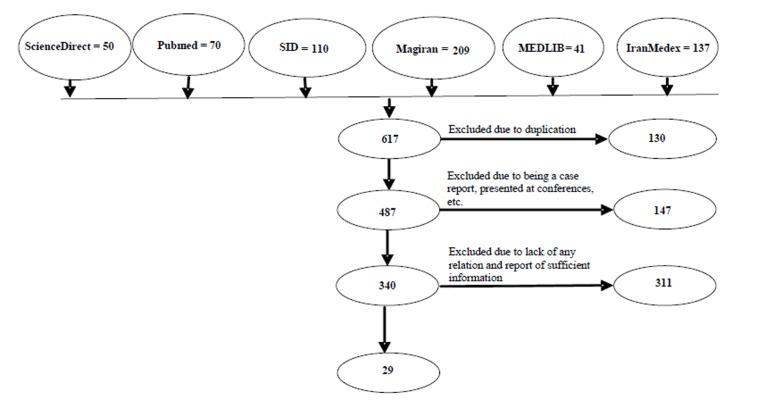
Flowchart of the study selection process.

In this systematic review study, the relevant data was collected using keywords including “drinking water fluoride,” “fluoride concentration,” “fluorosis,” “dent*”, “Iran*” and their Persian equivalents through PubMed, ScienceDirect, IranMedex, SID, MEDLIB, and Magiran databases. Articles published from 1990 to 2012, articles reporting fluoride concentration of drinking water and prevalence of fluorosis, and articles published in both Persian and English languages were regarded as inclusion criteria of the study. Articles presented at conferences as well as papers evaluating fluoride in resources other than water, e.g. tea, agricultural products, soil, etc., were excluded from the study. Following database searching, some authentic journals were manually searched in order to identify and cover more published articles. After excluding those articles relating weakly to the study objectives and selecting main articles, references of the selected articles were researched to being assured of identification and evaluation of the available articles and find more related articles. Out of 617 articles, 29 completely related articles were finally considered and the remaining not properly related to the study objecttives was excluded. The selected articles were completely and exactly evaluated (Figure 1). There were two articles published in English-language journals. Following extraction of the relevant data, they were initially summarized in extraction table and analyzed manually. Excel 2007 software was used to draw diagrams.

## Results

In this study, out of 617 articles, 29 articles completely related to the study objectives were finally selected, exactly studied, and the relevant data extracted (Tables 1 & 2).

**Table 1:** Extraction table for fluoride concentration of drinking waters in Iran

Authors/ year	City/ Province	Sampling method	Studied month/ season	Number/sampling resource	Maximum and minimum rate of water fluoride (ppm)
Azimi et al,^24^ 2000	Tehran	SPANDS	8 months: May-Dec.	2: Karaj & Jajroud rivers (river)	Jajroud (0.28-0.52), Karaj (0.15-0.35)
Hosseinpour Feizi et al,^25^ 2008-09	East Azerbaijan	SPANDS	—	668 urban and rural drinking water resources	0.1-2.8 with the mean of 0.26 ± 0.27
Mohseni Sajjadi et al,^26^ 2007-08	Arak	—	Nov. & Jun.	179 samples (agricultural well)	Nov. (0.03-0.53 with the mean of 0.3 ± 0.5), June (0.02-0.22 with the mean of 0.06 ± 0.03)
Sahargahi et al,^27^ 2001-10	Eslamabad Gharb	—	—	200 samples from urban and rural regions	0-0.9 with the mean of 0.32
Khademi & Taleb^28^	Isfahan	—	—	—	Mean in Najafabad:0.23, Jouzdan:0.6, Filvar:0.78, & Rahmatabad:1.35
Mirghaffari & Shariatmadari,^29^ 2003	Isfahan	—	Summer & Spring	—	Spring (0.09-0.4 with the mean of 0.3 ± 0.1), summer (0.01-0.14 with the mean of 0.05 ± 0.03)
Javan et al^30^	Boushehr	—	Mar. & Apr.	Drinking water of three schools	0.41, 0.46, 0.58
Rajaei et al,^31^ 2009-10	Birjand & Ghaen	—	Fall & Spring	54 samples from 27 stations	0.14-1.03 with the mean of 0.38
Shahriari et al,^32^ 2008	Southern Khorasan	—	—	314 samples from 7 towns	Mean in Birjand (0.47), Ghaen (0.59), Ferdows (0.50), Ferdows (0.53), Saraian (0.49), Sarbisheh (0.66), & Darmian (0.54), total mean=0.52
Poureslami et al,^33^ 2008	Kerman	—	—	42 samples from 8 big cities of Kerman province	Mean (SD) in Baft (0.41 ± 0.26), Kahlouj (0.44 ± 0.19), Sirja (:0.39 ± 0.039), Bam (0.43), Jiroft (0.34 ± 0.34), Zarand (0.47 ± 0.039), Rafsanjan (0.39 ± 0.026), Kerman (0.17 ± 0.034)
Basir et al,^34^ 2002	Khuzestan	SPANDS	—	8 cities: from three Maroon, Karoun, and Karkheh rivers	Maximum in Maroun (0.51), Karoun (0.31), & Karkheh (0.43)
Nasehinia & Nasseri,^35^ 2000-01	Damghan	SPANDS, ion electrode	High and low rain seasons (four seasons)	40 samples from 8 regions of city	Mean for low rain (0.37), high rain (0.6)
Dobaradaran et al,^19^ 2007	Boushehr	SPANDS	First half of the Iranian year	Ground waters of 13 villages of Borazjan	0.99-2.12 with the mean of 0.270
Ramezani et al,^36^ 2009	Zanjan	—	Summer	58 water reservoirs, 8 cities	Reservoirs: 0.002-0.574 with the mean of 0.06 ± 0.09 Tap water: 0.03-0.26 Abhar (0.26), Mahneshan (0.11), Soltanieh (0.11), Khoramdareh (0.09), Abbar (0.08), Khodabandeh (0.05), Heidaj (0.04), Zanjan (0.03)
Ramezani et al,^37^ 2009	Sari	—	Spring	34 drinking water reservoir of Sari	0.179-0.318 with the mean of 0.247 ± 0.031
Ramezani et al,^38^ 2009	Shiraz	—	Spring	36 drinking water reservoir of Shiraz	0.144-0.649 with the mean of 0.349 ± 0.153
Rahmani et al,^39^ 2008-09	Nourabad-e Mamasani	SPANDS	Oct. 2008 to Apr. 2009	7 regions of Mamasani town	Nourabad (0.4), Babamonir (0.31), Abpakhshan (0.53), Jouijan (0.31), Mouraki (0.41), Parin (0.3), Mirjan (0.1)
Davari et al^40^	Bastak, Hormozgan	—	—	9 samples from 3 regions of Bastak	Pond water of Harank 1 (0.8), pond water of Harank 2 (0.24), tap water of Harank (1.55), pond water of Bastak 1 (0.20), pond water of Bastak 2 (0.29), tap water of Bastak (0.85), tap water of Jenah 1 (0.76), tap water of Jenah 2 (0.75), pond water of Jenah (0.21)
Davari et al,^23^ 2002	Aghda,Yazd	SPANDS	—	10 samples from Aghda region	Well water of Aghda 1 (0.8), well water of Aghda 2 (0.44), well water of Aghda 3 (0.98), well water of Haftador 1 (1.3), well water of Haftador 2 (0.81), well water of Sarv Sofla 1 (0.85), well water of Sarv Sofla 2 (1.41), well water of Mazraeh No 1 (1.22), well water of Mazraeh No 2 (1.16), well water of Mazraeh No 3 (1.57),
Amouei et al,^41^ 2009-10	Khaf, Khorasan Razavi	SPANDS	Summer & Fall	32 water resources	N(Mean ± SD); Well: 25(0.90 ± 0.66)-Range(0.15–3.59) Spring:2(0.74 ± 0.32)- Range(0.32–1.10) Subterranean:4(0.62 ± 0.30)- Range(0.38–0.85) Total : 31(0.88 ± 0.62)- Range(0.11–3.06)
Moslemi et al^42^	Tehran	SPANDS	Summer & Winter	30- Karaj dam, Latian dam, Lar dam	Summer : Karaj dam (0.182), Latian dam (0.208), Lar dam (0.358) Winter : Karaj dam (0.129), Latian dam (0.194), Lar dam (0.305) Total: N (Mean ± SD); 30(0.229 ± 0.014)
Sepehri et al^43^	Kerman	—	—	16 regions of Kerman-well	0.154-0.341 with the mean of 0.195
Modabber Abbasi,^44^ 1991-92	Zanjan	SPANDS	22 Nov. 1991 — 21 Nov. 1992	2176 samples from 33 deep wells and 2 water reservoirs	0.349-0.731 with the mean of 0.561
Almasi et al,^45^ 1999	Kashan	SPANDS	Spring & Fall	201 samples from wells, springs, and ducts of Kashan	0.25-1.2, total mean: 0.653 ± 0.22 Mean of wells:0.7, springs:0.57, & ducts:0.49
Samarghandi & Sadri^46^ 1998-99	Hamadan & Bahar	-	—	150 samples from drinking waters of Hamadan & Bahar	Mean of Hamadan: 0.198, mean of Bahar:0.6
Araghizadeh et al,^47^ 2002	Bandarabbas	SPANDS	—	Sampling from drinking water of 16 regions	0.55-0.93 with the mean of 0.73
Shidfar et al,^48^ 2000	Ilam	—	Fall	96 samples from 4 regions of Ilam	Mean: 0.28, Oct.: 0.29, Nov.: 0.27, & Dec.:0.29

**Table 2:** Extraction table for prevalence of fluorosis in Iran

Author/year	City	Number & samples	Fluorosis
Khademi & Taleb^28^	Isfahan	254 elementary school students (12-7 years old)	Normal (37.38)38, suspicious (18.69)80, very mild (24.30)104, mild (17.29)74, moderate (6.7)17, severe (4.7)12
Araghizadeh et al,^47^2002	Bandarabbas	867 student from elementary schools (12-7 years old)	Normal (15)160, suspicious (23.2)59, very mild (31.1)79, mild (19.3)49, moderate (2.34)10, severe (0)0
Mortazavi et al,^49^ 2000	Deir- Boushehr	506 students (11-16 years old)	Girls= Normal (16.5), suspicious (35.10), very mild (25.8), mild (16.4), moderate (6.2), severe (0) Boys= Normal (12.1), suspicious (25.3), very mild (34.5), mild (18.5), moderate (9.6), severe (0)
Javan et al^30^	Boushehr	95 students (10-12 years old)	Normal (47.4)45, suspicious (16.8)16, very mild (12.6)12, mild (21.1)20, moderate (2.1)2, severe (0)0
Basir et al,^34^ 2002	Khuzestan	1152 students (12-15 years old)	Normal (48.52)559, suspicious (17.70)204, very mild (17.96)207, mild (10.50)121, moderate (4.68)54, severe (0.60)7 Maroon (1.36±1.32), Karoun (0.64±1.02), Karkheh (1.21±1.32)
Honarmand et al,^50^ 2006	Zahedan	334 students (7-10 years old)	Normal (69.16)231, suspicious (2.39)8, very mild (10.77)36, mild (8.39)28, moderate (8.39)28, severe (0.9)3
Mortazavi & Karimian,^51^ 1998-99	Jarghouieh Sofla & Olia	519 guidance school students	Jarghouieh Sofla: Normal (45.1), suspicious (19.1), very mild (10.2), mild (8.8), moderate (6.3), severe (0.8) Jarghouieh Olia: Normal (34.2), suspicious (22.1), very mild (19.8), mild (12.5), moderate (7.2), severe (1.8)

Time interval of the evaluated articles varied from 1991 to 2009. Generally, the studies covered 17 Iranian provinces. SPANDS sampling method was used to sample water in all studies within 236 months during the study period. It should be noted that 16, 12, 11, and 8 studies were conducted in spring, summer, fall, and winter, respectively. Resources used in 8, 17, 4, and studies included well water, urban and rural tap water, ground waters (spring and duct), and surface waters (river and pond). The evaluated studies considered 4434 samples of different water resources of the country. Also, 3727 students ranging 7-16 years old were studied to evaluate prevalence of fluorosis.

**Figure 2:**
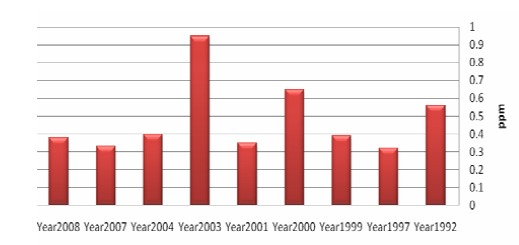
Mean fluoride concentration of different resources waters evaluated in the studies based on year.

To determine fluorosis severity, Dean Index was used in all studies. Figure 2 shows the mean fluoride concentration of waters in different evaluated resources based on the different years.

As seen, the highest rate of fluoride concentration of different water resources is attributed to the year 2002.

The average fluoride concentration of drinking waters was 0.43 ± 0.17 ppm with zero and 3.06 as its minimal and maximal values obtained for Kermanshah and Khorasan Razavi provinces, respectively.

**Figure 3:**
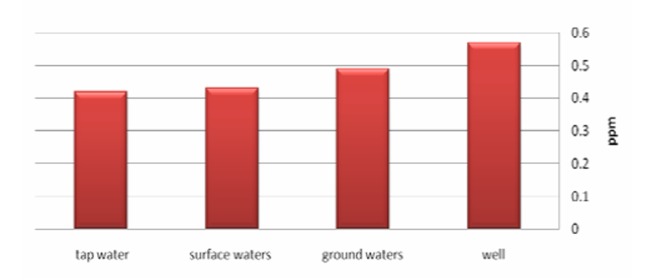
Mean fluoride concentration of different resources waters evaluated in the studies based on the understudy water resource.

**Figure 4:**
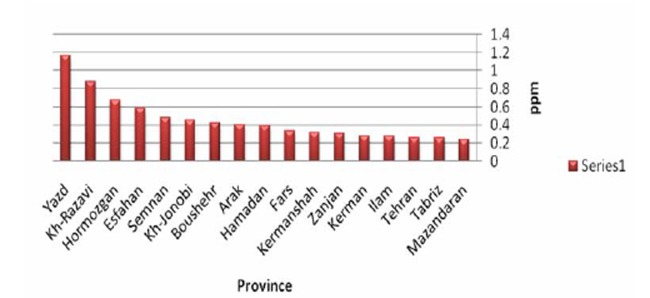
Mean fluoride concentration of different resources waters evaluated in the studies based on the understudy provinces.

Figure 3 demonstrates mean fluoride concentration of waters based on the different water resource. According to Figure 3, drinking waters of well has the highest mean concentration of the studied waters. The least one belongs to urban and rural tap waters. As mentioned, Iran has different ecological condifmean range of fluoride concentration in waters of provinces. Figure 4 demonstrates fluoride concentration of drinking waters in every province. As seen on the figure, Yazd and Mazandaran provinces have the highest and lowest rates of fluoride concentration. Tehran is regarded as a province with low fluoride concentration. As seen, only three provinces are at the standard range recommended by World Health Organization.

**Figure 5:**
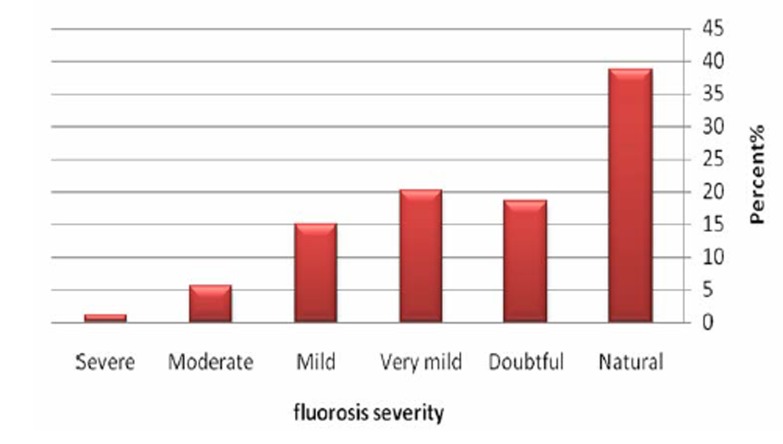
Prevalence of fluorosis based on its severity among the understudy samples in the evaluated studies

Figure 5 shows prevalence of fluorosis among samples of the evaluated studies based on its severity. According to Figure 5, there is normal fluorosis in about 39% of the samples and severe fluorosis was seen in only about 1% of the samples.

## Discussion

In 4434 samples evaluated in the conducted studies, average fluoride concentration was estimated 0.43 ± 0.17 ppm with zero and 3.06 as minimal and maximal values. The highest and lowest concentrations were seen in well, and urban and rural tap waters, respectively. Out of 3727 studied students, only 1% suffered from severe fluorosis.

Mean scores obtained from the studies conducted in Iran (0.43 ± 0.17 ppm) is less than standard values of 0.7 ppm for tropical and 1.2 ppm for cold regions recommended by World Health Organization. Therefore, appropriate plans and policies should be designed and executed to standardize fluoride concentration of different water resources of Iran. According to results of the study, most interventions should focus on increasing fluoride of drinking water since fluoride insufficiency leads to caries.^12^ Although fluoride concentration in drinking waters of Iran is less than standard values and their fluoride content should be increased, its disadvantages should also be considered in planning and policy makings, as increasing fluoride concentration above the permitted level would lead to undesirable disorders and complications such as mental disability, change of human chromosome structure, renal damages, and osteomalacia.^13-15^ Additionally, the results of global studies have demonstrated that the higher the fluoride concentration of drinking waters, the higher the prevalence rate of fluorosis in the population.^16-18^ According to recent reports provided by experts of Iran Fluoride Scientific Association, exceeding fluoride concentration of drinking waters from 0.7 mg/l may increase disadvantages rather than desirable effects of caries prevention.^19^ Reviewing studies conducted on measurement of drinking water fluoride in the US demonstrated that adding fluoride to drinking waters was not only useless but entailed certain disadvantages and economical costs.^20^ By precise evaluation of drinking water fluoride and its comparison with standards, authorities and experts should make appropriate decisions and offer proper plans to exactly adjust fluoride concentration of drinking waters.

Fluoride concentration of drinking waters of every province is demonstrated in Figure 4. According to this figure, fluoride concentrations of drinking waters of only three provinces are in accordance with standards defined by World Health Organization. Also, high fluoride concentration is seen in drinking water of provinces located at tropical regions where the water is mainly supplied by wells and ground waters. Necessary actions should be taken to reduce fluoride concentration in these regions. On the contrary, provinces located at cold and mountainous regions have low concentrations of fluoride. Water in these provinces is mainly supplied by surface and river waters. The regions require policies to increase fluoride level of drinking water.

In researches evaluated in this study, 3727 subjects were studied considering fluorosis. Generally, about 2274 cases (61%) suffered from different degrees of fluorosis. Of these, only 38 students (1%) experienced severe fluorosis. According to reports, the prevalence of fluorosis with fluoride concentration of 0.19 ppm was 47% in South Africa.^22^ Considering fluoride concentration found in the present study, it can be stated that the prevalence of fluorosis in South Africa is higher than Iran. The study conducted on 12-15 year-old students in Khuzestan reported prevalence rate of fluorosis about 51%, which is just below the average value calculated for the country. The results of the present study are in accordance with those of the Hormozgan province.^23^ Despite low fluoride concentration in drinking waters of Iran, high prevalence rate of fluorosis is seen in this study. Other resources including foodstuff, tea, and toothpastes may be regarded as the most important and justifiable sources of high fluoride dose thath individual receive. Further studies are warranted in order to identify main factors resulting in high prevalence of fluorosis.

Lack of meta-analyses of the extracted data due to different ways of data presentation is regarded as one of the drawbacks of the present study. It is recommended to use the same specific procedures in presenting the results of such studies. This was the reason why it was not possible to statistically evaluate the relationship between fluoride concentration and prevalence of fluorosis in this study.

## Conclusion

The results of the present study demonstrate that although average fluoride concentration in drinking waters of Iran is less than recommended standards, it is still higher than standard level in some regions. Generally, there are different concentrations of water fluoride in Iran due to variable ecological conditions of the country. Considering different effects of variable concentrations of fluoride, setting uniform policies for all regions of the country may result in undesirable complications. Therefore, appropriate policies should be made based on regional conditions as well as specific fluoride concentrations. Also, high prevalence of fluorosis in Iran was noted in spite of low fluoride concentrations, which may have resulted from fluoride uptake from other resources.
